# Topical Applications of a Novel Emollient Inhibit Inflammation in Murine Models of Acute Contact Dermatitis

**DOI:** 10.1155/2021/5594646

**Published:** 2021-04-13

**Authors:** Si Wen, Mengke Sun, Li Ye, Bin Yang, Lizhi Hu, Mao-Qiang Man

**Affiliations:** ^1^Dermatology Hospital of Southern Medical University, Guangdong 510091, China; ^2^Immunology Department, Key Laboratory of Immune Microenvironment and Disease (Ministry of Education), Tianjin Medical University, Tianjin 300070, China

## Abstract

The benefits of emollients for eczematous dermatitis and psoriasis have been thought to be due to the improvements in epidermal function, including epidermal permeability barrier, stratum corneum hydration, and stratum corneum pH. We determined here whether emollient can direct inhibit cutaneous inflammation. Ear inflammation was induced by topical application of either 12-O-tetradecanoylphorbol-13-acetate (TPA) or 1-fluoro-2,4-dinitrofluorobenzene (DNFB). Either 1% hydrocortisone cream or the novel emollient was applied to the right ear of the mice 45 min and 2 hours after TPA or DNFB application. The untreated left ear served as untreated controls. Both ear weight and ear thickness were measured 24 hours after TPA and DNFB application. Topical applications of either hydrocortisone cream or emollient significantly decreased both ear thickness and ear weight in comparison to untreated controls. In DNFB model, hydrocortisone significantly lowered expression levels of mRNA for IL-1*α*, IL-1*β*, and TNF*α*, while the emollient markedly decreased expression levels of IL-1*α* and TNF*α* mRNA. In TPA model, both hydrocortisone and emollient significantly decreased expression levels of IL-1*α*, IL-1*β*, IL-6, and TNF*α* mRNA. In parallel, inflammatory infiltration was also reduced by topical applications of either hydrocortisone or emollient. These results demonstrate that this novel emollient can directly inhibit cutaneous inflammation in murine models of both acute irritant contact dermatitis and acute allergic contact dermatitis. However, whether this emollient could also alleviate eczematous dermatitis in humans remains to be explored.

## 1. Introduction

Eczematous dermatitis is a common skin disorder, with prevalence of 10% in adult and as high as 24% in children aged 6-7 years [1, 2]. Although glucocorticoids and immunosuppressants can alleviate eczematous dermatitis, frequent relapse requires long-term treatment, leading an increase in incidence of adverse reactions. However, a number of studies have shown that topical emollients benefit dermatitis although some studies showed no benefits in infants [3–5]. For example, topical emollient alone can improve clinical signs and symptoms of atopic dermatitis [6–8]. The efficacy of emollient is comparable to that of hydrocortisone for atopic dermatitis in humans [9]. Moreover, daily applications of emollient for 6 months can reduce the risk of atopic dermatitis by 50% in infants [10]. Similarly, Horimukai et al. [11] showed that topical emollient reduced the incidence of atopic dermatitis by 32% in infants. Furthermore, topical emollients not only improve atopic dermatitis but also prevent atopic flare by 41% and delay the flare by 152 days [12]. Additionally, combination of emollient and glucocorticoids can reduce the usage of glucocorticoids, enhance efficacy, and delay relapse in comparison to glucocorticoids alone [8, 13–15]. Finally, combination therapy of emollient and glucocorticoids increases efficacy for psoriasis, another common inflammatory skin disorder [16–18]. A recent study showed that topical emollient alone delays the relapse of psoriasis [19]. Collectively, this evidence indicates that emollients can alleviate cutaneous inflammation.

Regarding the mechanisms by which emollients improve cutaneous inflammation, it is assumed to be primarily due to the improvements in epidermal permeability barrier function and stratum corneum hydration. Indeed, dysfunction in epidermal permeability barrier provokes cutaneous inflammation and predisposes to the development of cutaneous inflammation [20–24]. Likewise, either reduction in stratum corneum hydration or elevation in stratum corneum pH can also induce or exacerbate cutaneous inflammation [25]. Conversely, acidification of the stratum corneum prevents the development of atopic dermatitis in mice [26, 27]. Thus, emollient-induced improvements in epidermal function can contribute to the alleviation of cutaneous inflammation. However, some emollients contain fatty acids, which can directly inhibit cutaneous inflammation, independent of either epidermal permeability barrier or stratum corneum hydration [28]. In the present study, we determined whether topical applications of a novel emollient containing natural oils can inhibit cutaneous inflammation in mouse models of acute contact dermatitis.

## 2. Materials and Methods

### 2.1. Experimental Design

#### 2.1.1. Animals and Materials

Eight-week old female C57BL/6J were purchased from Guangdong Animal Center (Guangzhou, Guangdong, China) and were fed with mouse diet and water *ad libitum*. Emollient, YuZe Skin Barrier Recovery Body Lotion®, was provided by Jahwa United Company (Shanghai, China). Main ingredients in the emollient include Glycerin, Olea europaea (olive) fruit oil, Carthamus tinctorius (safflower) seed oil, Butyrospermum parkii (shea butter), Persea gratissima (avocado) oil, Oryza sativa (rice) bran oil, sodium stearoyl lactylate, and sodium methyl stearoyl taurate. Hydrocortisone cream (1%) (Tianjin pharmaceutical, China) was purchased from local pharmacy store. Both 12-O-tetradecanoylphorbol-13-acetate (TPA) and 1-fluoro-2,4-dinitrofluorobenzene (DNFB) were purchased from Merck (Darmstadt, Germany) and Aladdin (Shanghai, China), respectively.

#### 2.1.2. Experimental Protocols

All animal procedures were approved by the Animal Study Subcommittee of the Southern Medical University and performed in accordance with their guidelines. Anti-inflammatory assay was carried out according to the method previously described with slight modification [29, 30]. Irritant contact dermatitis was induced by a single topical application of 10 *μ*l of 0.03% (wt/vol. in acetone) TPA on the inner and outer surfaces of both ears of mice. Allergic contact dermatitis was induced by single topical application of 20 *μ*l of 0.35% DNFB to the inner and outer surfaces of both ears of mice 5 days following sensitization with 25 *μ*l of 0.5% DNFB. The right ears were treated with either 1% hydrocortisone or emollient at 45 min and 2 h following application of TPA or DNFB while the left ears served as untreated controls. Additional group of normal mice served as normal controls. Twenty-four hours after TPA and DNFB application, ear thickness was measured with a digital caliper (Mitutoyo Corp., Tokyo, Japan), followed by 6 mm full skin biopsies for measurement of ear weight. Afterwards, mice were euthanized with overdose of isoflurane, and ear tissue samples were taken and fixed with 4% formaldehyde in phosphate-buffered saline (PBS) and embedded in paraffin. Ear thickness and weight were expressed as percentage of normal controls.

#### 2.1.3. Q-PCR for mRNA Expression

For analysis of the mRNA expression, ear samples were taken after the measurement of ear thickness and weight. Total RNA was isolated from whole ear, using TRI Reagent (Sigma). First strand cDNA was synthesized from 1 *μ*g of total RNA with the PrimeScript RT reagent Kit (cDNA Synthesis Kit) (Takara Bio, Japan). The expression levels of mRNA for proinflammatory cytokines were determined by qPCR. The real-time PCR contained 20 ng of reversed transcribed total RNA, 450 nM forward and reverse primers, and 10 *μ*l of 2x LightCycler 480 SYBR Green I Master in a final volume of 20 *μ*l in 96-well plates using Bio-Rad CFX96 connect Real-Time PCR System (Bio-Rad, California, USA). Quantification was performed by the comparative C_T_ method with mouse GADPH used for normalization. Primer sequences are listed in supplemental Table 1. The relative expression of the mRNAs compared to mRNA in normal mice was calculated. Data are expressed as percentage of normal controls (setting normal controls as 100%) [31].

#### 2.1.4. Statistics

Data are expressed as the mean + SEM. The GraphPad Prism 5 software (GraphPad Software, La Jolla, CA, USA) was used for all statistical analyses. One-Way ANOVA with Tukey correction was used to determine the significances when three or more groups were compared. Unpaired two-tailed student's *t*-test with Mann–Whitney test correction was used to determine the statistical significances when two groups.

## 3. Results

**Figure 1:**
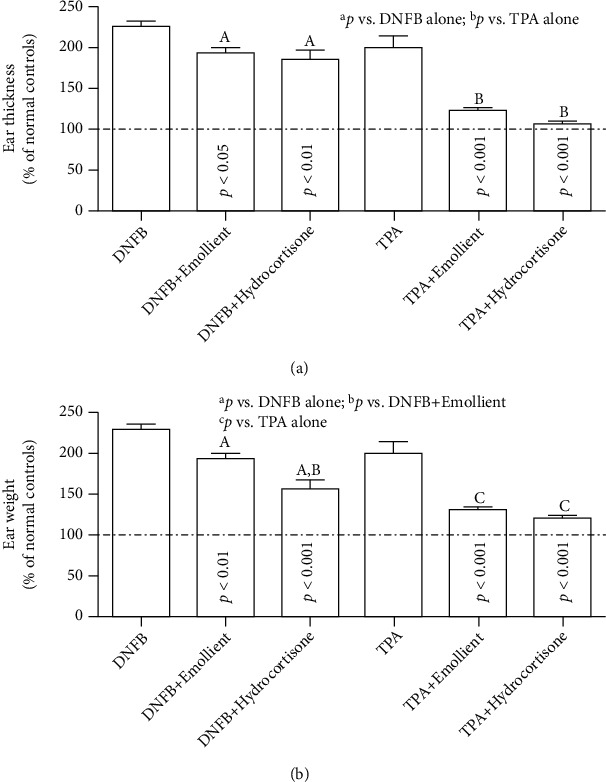
Topical emollient decreases ear weight and thickness in murine models of acute contact dermatitis: (a, b) depict ear thickness and weight, respectively. Data are expressed as % of normal controls, setting normal controls as 100%. Significances are indicated in the figures. *N* = 5 for all in irritant contact dermatitis model. In allergic contact dermatitis model, *N* = 6 for normal controls and *N* = 7 for all others.

**Figure 2:**
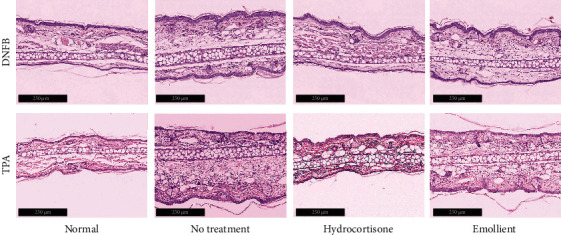
Histology of the skin. Skin samples were taken immediately after measurement of ear thickness as described in Materials and Methods. Scale bar = 250 *μ*m for all.

We first measured ear thickness and weight, indicators of ear swelling. As shown in Figure 1, topical application of either TPA or DNFB markedly increased both ear thickness (Figure 1(a); *p* < 0.001 normal vs. either TPA or DNFB alone) and ear weight (Figure 1(b); *p* < 0.001 normal vs. either TPA or DNFB alone). Both topical emollient and hydrocortisone significantly reduced ear thickness and ear weight in both TPA and DNFB models. Notably, the magnitude of reductions in ear thickness was comparable between emollient and 1% hydrocortisone in both TPA and DNFB models (Figure 1(a)). Similarly, the extent of reduction in ear weight did no differ significantly between emollient and hydrocortisone in TPA model, while ear weight in hydrocortisone-treated group was lower than that in the emollient-treated group in DNFB model (Figure 1(b); *p* < 0.05). Emollient-induced reductions in ear thickness and ear weight were accompanied with decreased inflammatory infiltration (Figure 2). These results indicate that this novel emollient and hydrocortisone exhibit comparable efficacy in reductions in ear swelling in murine models of acute contact dermatitis.

**Figure 3:**
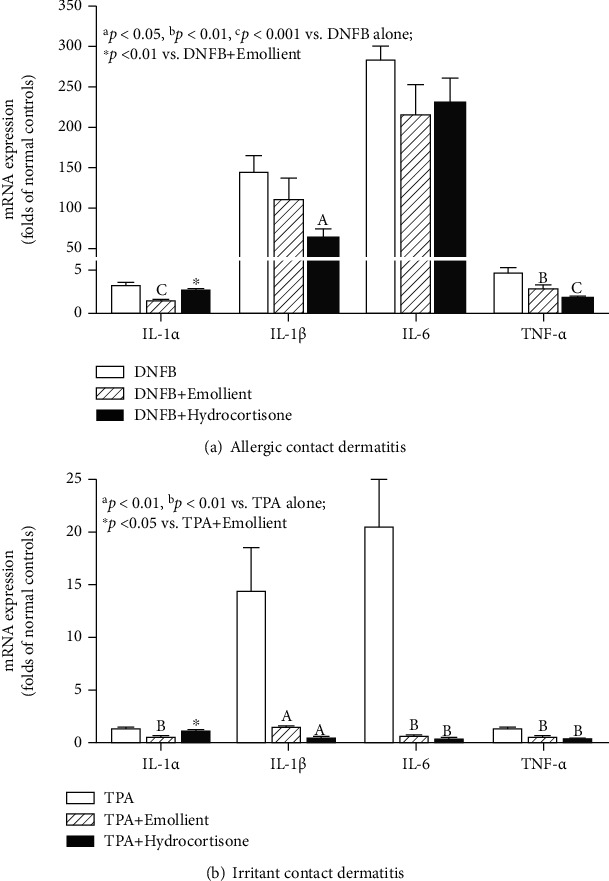
Topical emollient decreases expression levels of mRNA for proinflammatory cytokines: (a, b) display expression levels of mRNA for proinflammatory cytokines in allergic and irritant contact dermatitis model, respectively. Data are expressed as folds of normal controls, setting normal controls as 1. Significances are indicated in the figures. *N* = 5 for all in irritant contact dermatitis model. In allergic contact dermatitis model, *N* = 6 for normal controls and *N* = 7 for all others.

We next assessed the expression levels of mRNA for proinflammatory cytokines. In allergic contact dermatitis model, DNFB treatment increased expression levels of mRNA for proinflammatory cytokines (Figure 3(a); DNFB alone vs. normal controls *p* < 0.001 for all cytokines). Treatment with hydrocortisone significantly lowered expression levels of IL-1*α*, IL-1*β*, and TNF*α* but not IL-6. Expression levels of mRNA for IL-1*α* and TNF*α* were also significantly decreased following the treatment with the emollient, without significant changes in expression levels of IL-1*β* and IL-6. In irritant contact dermatitis model, expression levels of mRNA for IL-1*α*, IL-1*β*, and IL-6 were also markedly elevated following TPA treatment, but the levels of TNF*α* mRNA were not increased significantly (Figure 3(b); TPA alone vs. normal controls, *p* < 0.05 to *p* < 0.001). Treatment with emollient lowered expression levels of mRNA for all four cytokines in TPA model. Surprisingly, 1% hydrocortisone did not reduce the expression levels of IL-1*α* mRNA. Together, these results demonstrated that topical applications of this novel emollient inhibit cutaneous inflammation in mouse models of acute contact dermatitis.

## 4. Discussion

Eczematous dermatitis is a common skin disease. Currently, glucocorticoids and immunosuppressants are the primary modalities in the treatment of this disorder. Although these products are effective, adverse reactions limit their use. Evidence indicates that emollients can be used as adjuvant interventions in the management of eczematous dermatitis [4, 8, 32, 33]. We showed here that a novel emollient inhibits acute contact dermatitis in mice. It has been presumed that the benefits of emollients for eczematous dermatitis are attributable to the improvements in epidermal permeability barrier and stratum corneum hydration [34–36]. This presumption can hold true in chronic dermatitis with defective epidermal permeability barrier and reduced stratum corneum hydration. However, both transepidermal water loss rates and stratum corneum hydration levels are normal in acute dermatitis [37], suggesting that other mechanisms contribute to the inhibition of inflammation by this novel emollient. One potential mechanism can be due to activation of peroxisome proliferator-activated receptors (PPAR) *α* and *γ* because this novel emollient contains linolenic acid- and linoleic acid-enriched natural oils such as Olea europaea oil, Carthamus tinctorius seed oil, and Persea gratissima oil. Previous studies showed that linoleic acid can activate both PPAR*α* and *γ* [38], while linolenic acid activates PPAR*γ* [39]. Topical applications of either PPAR*α* or *γ* ligands inhibit cutaneous inflammation in PPAR receptor-dependent manner in acute models of contact dermatitis [28–30]. Thus, inhibition of inflammation by this novel emollient can be ascribable to its activation of PPAR*α* and *γ*.

While glucocorticoids can effectively improve eczematous dermatitis, these products can also compromise epidermal function such as disruption of epidermal permeability barrier function, skin atrophy, and reduction in antimicrobial peptide expression [40–42]. Previous studies demonstrated that topical PPAR ligands prevent glucocorticoids-induced alterations in epidermal proliferation and permeability barrier function via upregulation of epidermal differentiation and keratinocyte proliferation in normal mouse skin [43]. PPAR*α* activator alone alleviates cutaneous inflammation in murine model of atopic dermatitis [44]. Combination of glucocorticoids and the PPAR*α* activator exhibits superior efficacy to glucocorticoids alone in inhibition of cutaneous inflammation in murine model of atopic dermatitis, while preventing the emergence of glucocorticoids-induced epidermal side effects [45]. Collectively, a line of evidence suggests a potential utility of emollients containing PPAR ligands in the management of inflammatory dermatoses.

In conclusions, topical application of a novel emollient inhibits cutaneous inflammation in murine models of acute contact dermatitis. Because of the inhibitory effects of this novel emollient on cutaneous inflammation, this novel emollient (possibly other comparable emollients) alone or in combination with glucocorticoids could be a valuable approach in the management of acute and chronic eczematous dermatitis. However, the benefits of this novel emollient for eczematous dermatitis in clinical setting remain to be explored.

## Data Availability

The data that support the findings of this study are available upon reasonable request.

## Supplementary Materials

Supplementary Materials Table S1: the primers for QRT-PCR analyses.
