# Supplementary material for: Topical Applications of a Novel Emollient Inhibit Inflammation in Murine Models of Acute Contact Dermatitis
Source: Biomed Res Int. 2021 Apr 13;2021:5594646. doi: 10.1155/2021/5594646 (PMC8057889; doi:10.1155/2021/5594646)
Supplement: Supplementary Materials — Table S1: the primers for QRT-PCR analyses. [file 5594646.f1.pdf]

Table S1. The primers for QRT-PCR analyses.

Gene	Forward primer(5’-3’)	Reverse primer(5’-3’)
GAPDH	ACCTGCCAAGTATGATGACATCA	GGTCCTCAGTGTAGCCCAAGAT
IL1 α	TCTGCCATTGACCATCTC	ATCTTCCCGTTGCTTGAC
IL1 β	GAAATGCCACCTTTTGACAGTG	TGGATGCTCTCATCAGGACAG
IL6	TAGTCCTTCCTACCCCAATTTCC	TTGGTCCTTAGCCACTCCTTC
TNF α	CAGGCGGTGCCTATGTCTC	CGATCACCCCGAAGTTCAGTAG
